# Simulated learning in musculoskeletal assessment and rehabilitation education: comparing the effect of a simulation-based learning activity with a peer-based learning activity

**DOI:** 10.1186/s12909-014-0253-6

**Published:** 2014-11-29

**Authors:** Mark Hecimovich, Simone Volet

**Affiliations:** School of Psychology and Exercise Science, Murdoch University, Murdoch, Western Australia; School of Education, Murdoch University, Murdoch, Western Australia

**Keywords:** Standardised patient, Musculoskeletal, Exercise physiology, Clinical skills, Clinical knowledge, Motivation for lifelong learning

## Abstract

**Background:**

Musculoskeletal disorders and diseases are leading causes of pain, physical disability, and doctor visits throughout the world. Health professionals must be trained to assess, treat through rehabilitation and monitor patients with these disorders. Yet, due to overcrowded curricula, some health education programs struggle to accommodate more than minimal training in musculoskeletal conditions. Consequently, educators in these professions must consider how traditional instruction could be complemented effectively to enhance students’ preparation for the diverse musculoskeletal disorders and pathologies they may encounter. The purpose of this study was to explore the benefits that can be obtained from laboratory practice in musculoskeletal conditions with a standardised patient, rather than a peer patient, in a condensed time frame.

**Methods:**

Two groups of students were assigned to either a standardised or a peer patient condition for 2 × 2 hours musculoskeletal assessment and rehabilitation lab sessions. All students completed a pre-post matched questionnaire measuring their clinical knowledge, confidence in clinical skills and motivation for further learning. Their clinical skills were tested at the end. Students and standardised patients’ perceptions of the simulated learning environment to practise musculoskeletal assessment and rehabilitation were also elicited.

**Results:**

A t-test for independent samples revealed that students working with standardised patients displayed significantly higher standards of practical clinical skills than those working with peer patients (p=0.018). Using MANOVAs with repeated measures, no interaction effect for clinical knowledge, confidence in clinical skills, and motivation for future learning were found, both groups displaying significantly enhanced cognition and motivation. Three positive and two negative themes emerged from the analysis of students’ perceptions of the simulated learning environments. These were consistent with the simulated patients’ perceptions.

**Conclusions:**

The findings of this study provide support for the value of using standardised patients to enhance clinical skills in musculoskeletal assessment and rehabilitation when the timeframe for laboratory practice is limited. Students’ perceptions of their experience contributed to explain why confidence in clinical skills might not necessarily improve when practising with standardised patients. Suggestions are made for optimising learning with standardised patients and for addressing the economic challenge on health education programs of hiring standardised patients.

## Background

Musculoskeletal disorders and diseases are a leading cause of pain, physical disability, and doctor visits throughout the world [1]. Therefore it is important that health professionals are optimally trained to assess (or comprehend basic assessments completed on a referred patient), treat through rehabilitation and monitor patients with these disorders. Health professions, which commonly see patients with these disorders, include exercise physiology, physical therapy (physiotherapy), medicine, athletic training (athletic therapist), chiropractic, occupational therapy, and osteopathy.

One of the major challenges for curriculum developers in the health professions is that the musculoskeletal system has multiple varying conditions, injuries and pathology [2]. Any health education program would struggle to cover all aspects in one rehabilitation unit or module. The training and curriculum of the different related professions, therefore, vary substantially in their musculoskeletal content. For example, physical therapy [3], athletic training [4] and chiropractic [5] curricula dedicate more hours and in greater depth and detail to this area than, for example, medicine [2] and exercise physiology [6]. This means that students in some programs are better prepared than others to perform these skills safely [7]. As a consequence, it is vital for educators in these professions to consider how traditional forms of instruction could be complemented to best prepare students for the diverse range of clinical conditions they may encounter in their professional practice. Most importantly, they should be sensitized to the uniqueness of each condition and be fully committed to engage in continuous professional learning in this area.

The inadequacy of the medical curriculum to address musculoskeletal disorders and diseases has long been recognised and has received increased attention in the last decade. Many countries have adopted different approaches to addressing this problem. In the United States, one of the first unified attempts at undergraduate musculoskeletal curriculum reform started with the establishment of the National Bone and Joint Decade [8]. One area of focus was to ensure dedicated instruction in musculoskeletal medicine in 100% of U.S. medical schools (Project 100), an objective that was addressed in a number of ways [9] including re-evaluation of musculoskeletal curricula by schools and accreditation bodies (Contemporary issues in medicine report VII).

A few studies also attempted to compare different types of musculoskeletal learning instruction in medical education. For example de Jong et al. [10] randomised students to small group tutorials or large group interactive seminars for cognitive instruction, and found no differences in end-of-sequence test scores, but greater satisfaction with the small group format. Modica et al. [11] compared large group lectures and physical examination demonstration with a web-based tutorial, and found no differences in exam or OSCE performance whilst Vivekananda-Schmidt et al. [12] found that addition of a computer-based module to an existing curriculum resulted in improved OSCE results.

Interestingly, while the use of standardised patients (SPs) is frequently utilised in the teaching and assessment of physical exam or diagnostic skills in medical education [13], this form of instruction is still limited in the area of musculoskeletal assessment and rehabilitation education. An SP is defined as a person who repeatedly portrays his or her diagnosis or a set of symptoms, or a healthy individual who has been carefully coached to accurately portray a specific patient diagnosis or set of symptoms [14]. Initially pioneered in medicine in the 1970s in response to lack of availability of ‘real patients’ for ward-based teaching, and the recognition that students required more opportunities to practice in a controlled environment prior to actually being released in a clinical setting [15,16], the use of actors to act as patients has become commonplace in many health profession education programs [17]. Extending its use to musculoskeletal assessment and rehabilitation in medical education may therefore be beneficial [2].

The value of simulated learning in preparing students for clinical encounters related to musculoskeletal assessment and rehabilitation in programs which do not have multiple units in this area, such as medicine [2] and exercise physiology [6] needs to be examined. Whilst also aiming at knowledge and skill development, the use of SPs provides vital professional preparation to complement traditional instruction. According to Collet et al. [18], it should target the preclinical and trainee stage levels, when performance-based teaching and assessment becomes critical.

Performance-based teaching and assessment are terms used to describe methods that allow educators to focus on clinical skills rather than simply clinical knowledge. Whilst traditional methods (ranging from lectures to case-based multiple-choice tests) may be effective for teaching and testing knowledge, fostering students’ development of clinical skills is not as amenable to these methods [19]. Performance-based approaches provide opportunities to teach and test at the same time the amalgam of knowledge, skills, and attitudes that are integral to health professionals’ work [15,16]. Particularly in the context of communicative and cultural competencies, performance-based methods possess intrinsic advantages over traditional methods since they require both declarative knowledge and procedural knowledge [20].

A number of empirical studies have revealed that students trained in certain medical procedures with SPs showed improved skills compared to students in a traditional training program, as well as increased confidence in that procedure [21-23]. Survey studies also revealed reduced stress and anxiety in students performing examinations, and increased satisfaction and attention to patients’ feelings, integrity, and privacy [24]. In all these studies, the outcome measures were mostly an isolated aspect of one of the skills, or an estimation of feelings such as self-confidence or anxiety of the students in either male or female patients. A number of studies have demonstrated the pedagogical advantages for using SPs to educate medical students [25-27] and as a result, SPs are commonly used in medical education and the American Physical Therapy Association (APTA) has included the use of standardised patients (SPs) as part of its educational strategic plan [28]. The most frequently cited advantages include the opportunity to create and expose learners to complex cases [26], directly test newly acquired knowledge of patient- interviewing skills [25], and explore communication challenges inherent to complex patient care [27,29].

The present study aimed to contribute to this body of research by examining the benefits of training practice in musculoskeletal assessment and rehabilitation using SPs, and in a condensed time frame. Exploring the benefits that can be obtained from SPs instruction in a condensed time frame is particularly important in areas of health professional education, which cannot incorporate multiple musculoskeletal assessment and rehabilitation units or modules.

The study endeavoured to compare the effect of a simulation-based learning activity (involving actors as patients) with a peer-based learning activity (peers acting as patients for each other) on a range of clinical, cognitive and motivational outcomes. This included students’ practical clinical skills, their clinical knowledge, their confidence in evaluating a shoulder pathological condition, as well as their motivation and desire to learn about musculoskeletal rehabilitation related to other conditions in the future.

Four hypotheses were generated:

1. It was hypothesised that after practice, the group of students in the simulated learning environment involving actors would display higher standards of practical clinical skills in comparison to the group of students in the peer-based learning environment, because the actors as SPs would be responding (as instructed) to assessment and rehabilitation procedures, therefore students would get more realistic responses and learn from these.
2. No group differences were expected in the development of clinical knowledge on the ground that such knowledge could be acquired through lecture instruction.
3. Based on prior research, it was hypothesised that confidence in clinical skills would be higher for the group of students who practised their skills with an actor acting as patient rather than a peer as patient; on the ground that practising with a SPs would boost their confidence in dealing with “real” patients.
4. Finally, it was also hypothesised that the simulated learning environment would produce greater motivation for further learning, on the ground that the experience of a patient’s unique reactions to a simulated condition would lead to a realisation of the complexities encountered in musculoskeletal rehabilitation and therefore the need for further learning.

## Methods

### Participants and study modules

Participants were 3^rd^ year university exercise physiology students (N = 43), all volunteers, who were about to enter their year-long clinical practicum. Participants had no previous coursework in shoulder evaluation and musculoskeletal rehabilitation. This research was conducted following ethics approval from the University’s Human Research Ethics Committee, and student written consent. Exercise physiology students were ideal candidates because in the country where the study was conducted, exercise physiologists are allowed to administer musculoskeletal exercise rehabilitation to patients in a variety of clinical settings.

The study module on shoulder evaluation and musculoskeletal rehabilitation, designed for this study, took place over two weeks, with the final testing on the following week. Each week, participants attended a 2-hour didactic lecture, followed by a 2-hour lab offering opportunities to practise shoulder evaluation and management, on the specific topics presented in the preceding lecture. Students were assigned to either the simulated learning environment (actors as standardised patients) or the peers’ learning environment (peers acting as patient for each other) for their lab practice in the first week. The results of a pre-test were used to form two groups with comparable entry profiles.

### Assessment instruments

#### Clinical knowledge (pre-post)

A new 10-item written test (Table 1) was developed to assess students’ clinical knowledge about shoulder anatomy, evaluation and rehabilitation management, and more specifically, the frequently encountered shoulder conditions, rotator cuff tendinopathy/subacromial (RCT) impingement syndrome pathology and multidirectional instability, which were targeted in the lectures and labs.

**Table 1:** **Clinical knowledge pre-post short answer questions**

	**Question**
1.	Please list the four muscles which comprise the rotator cuff:
2.	Please list the range of motions (ROM) normally assessed on the shoulder:
3.	Please name the orthopaedic test which pushes the supraspinatus tendon against the anterior surface of the corocoacromial ligament with patient sitting or standing, shoulder flexed to 90 degrees and then internal rotated without resistance by the patient:
4.	The sulcus sign/inferior test is use to determine which shoulder pathology:
5.	Scapulohumeral rhythm consists of integrated movements of which areas of the shoulder complex:
6.	Early exercise interventions concentrate normally of 3 areas. Please list 1 of these areas:
7.	The application of alternating isometrics and rhythmic stabilization techniques is designed to develop strength and stability of proximal muscle groups in response to shifting loads. Please briefly outline what is meant by this:
8.	It is imperative that the proximal stabilizing muscles of the thorax, neck, and scapula function properly before initiating dynamic strengthening of the muscles that move the glenohumeral joint through the ROM to avoid faulty mechanics. Please briefly outline what is meant by this:
9.	As soon as the patient develops control of scapular and humeral motions and the basic components of the desired activities without exacerbating the symptoms, you need to initiate specificity of training toward the desired functional outcome by progressing the strengthening exercises to maximum resistance concentrically and eccentrically. Please provide a summary of what this concept entails:
10.	The shoulder girdle functions in both open- and closed-chain activities, and therefore the muscles should be trained to respond to both situations. Why is this important:

#### Self-confidence (pre-post)

A 9-item self-confidence scale (Table 2) was developed to assess students’ confidence in their knowledge and skill in assessing and initiating beginning exercise techniques for RCT pathology and shoulder multidirectional instability. Currently no validated measure of shoulder self-confidence exists. The new instrument used a format similar to the general Clinical Skills Confidence Questionnaire developed and validated by the researchers [30].

**Table 2:** **Self-confidence in clinical skills pre and post test**

		**Not at all confident**		**↔**		**Highly confident**	
**1**	How confident are you in doing basic shoulder assessments such as muscle testing and range of motion on a person whom you are not familiar with?	1	2	3	4	5	6
**2**	How confident are you in being able to make a patient comfortable, minimising their pain and anxiety, whilst performing a shoulder assessment on your patient?	1	2	3	4	5	6
**3**	How confident are you in taking a history on a patient with rotator cuff tendonitis/impingement syndrome?	1	2	3	4	5	6
**4**	How confident are you in performing shoulder palpation on a patient with shoulder complaints?	1	2	3	4	5	6
**5**	How confident are you in interpreting a patient’s verbal and physical response to orthopaedic assessment procedures? In other words, interpreting if the assessment is positive or negative for a condition such as facet or impingement syndrome?	1	2	3	4	5	6
**6**	How confident are you in explaining to a patient what may be causing their pain during a certain procedure; for example, shoulder pain during a specific orthopaedic procedure	1	2	3	4	5	6
**7**	How confident are you at performing a basic shoulder assessment which includes observation, orthopaedic, muscle testing, and range of motion procedures?	1	2	3	4	5	6
**8**	How confident are you in interpreting the findings of an active function tests or either the spine or shoulder?	1	2	3	4	5	6
**9**	How confident are you at initiating a shoulder range of motion and strengthening exercise technique?	1	2	3	4	5	6

#### Engagement in lifelong professional learning in exercise physiology (pre-post)

A new 3-item scale (Table 3) was developed to assess students’ motivation and desire to learn more about musculoskeletal rehabilitation related to other conditions in the future, and for professional lifelong learning more generally. This measure was accompanied by an open-ended question to provide students with an opportunity to elaborate on their answers.

**Table 3:** **Engagement in lifelong learning**

		**Not very often**				**Extensively, very all the time**	
**1**	I look up for resources about the way exercise physiology or exercise science practices are conducted in addition to what is required in my units	1	2	3	4	5	6
	Please explain:						
**2**	I seek opportunities to develop my practical skills through volunteering for staff research or community projects.	1	2	3	4	5	6
	Please explain:						
**3**	I am open to new learning opportunities in the field of exercise physiology or exercise science.	1	2	3	4	5	6
	Why?						
	What kind?						

#### Clinical skills (post)

This involved a practical test of students’ capacity to evaluate a shoulder for the RCT and multidirectional instability condition. After this assessment, students in the SPs condition were invited to answer a few additional open-ended questions eliciting their own assessment of their learning in their assigned practice-training environment.

#### Standardised-patient and peer-patient student evaluation (post)

Standardised and Peer-patients were invited to answer an open-ended question on the performance of the student during the clinical skills test.

### Procedures

Results from the initial clinical knowledge test were used to assign students to one of the two groups for the laboratory sessions, in such a way that each group had a comparable aggregate entry profile in terms of students’ prior knowledge in RCT and multidirectional instability pathology. 22 students were assigned to a Peer Patient (PP) condition (16 Male, 6 Female; 18 aged between 18-22 years, 3 between 23-28 years, and 1 older than 28 years) and 21 to a Standardised Patient (SP) condition (11 Male, 10 Female; 16 aged between 18-22 years, 4 between 23-28 years, and 1 older than age 28 years). Each week, over the course of two weeks, both groups attended a two-hour lecture (same for both groups). This lecture provided background on the shoulder conditions, including pathomechanics, signs and symptoms, evaluation techniques and rehabilitation protocols related to the practice-training activity, or lab. Following the lecture, students joined their assigned training practice group, either *a simulated learning environment (with a standardised patient)* or *a peer-based learning environment (peers acting as patients for each other).* Students had an opportunity to practise their skills with, respectively, a different peer or different actor. The task, the room and the duration of the laboratory session, for students to practise their skills in evaluating and managing RTC pathology, were the same for both groups. One lecturer in exercise physiology with expertise in rehabilitation was present in each session but did not intervene.

One week after the completion of the two-week lecture and lab sessions, all participants returned to complete the post-tests. These included the same knowledge and confidence tests as at the beginning. In addition, students’ clinical skills were also assessed with a practical test, performed with respectively, a peer or standardised patient, depending on the group they had been assigned to. Students in the SPs condition were invited to answer a few additional open-ended questions eliciting their own assessment of their learning in their assigned practice-training environment. SPs and PPs were invited to answer an open-ended question eliciting their own assessment on the performance of the student being assessed during the practical skills test.

### Standardised patients

A pool of 22, 5^th^-year, clinical-based chiropractic student volunteers were trained as standardised patients. These volunteers were from a chiropractic program offered in a separate school, which means it was highly unlikely that they would know any of the participants. These volunteers were considered ideal candidates to act as SPs in that they possessed an excellent understanding of the shoulder region and the various evaluations that they would be exposed to as a standardised patient. However, they were instructed to provide constructive feedback to students only on their professional and communication skills, and not on how well they performed the skill. This was done to create a more real life situation in which patients usually are unfamiliar with how well a specific skill is being performed. Their age range spanned from 22 to 40 years of age. All the standardised patients were required to attend two preliminary training sessions on shoulder examination procedures and their responsibilities as a standardised when the student was performing an evaluation on them, and when requests made of them (i.e., lift your arm up and to the side). Once the actual lab commenced the SPs responded according to the specific evaluation technique the student was performing.

### Data analysis

The reliability of the two scales on each occasion was determined using Cronbach’s alphas: 9-item Confidence scale (t1 α = .94; t2 α = .93); and 3-item Engagement scale (t1 α = .72; t2 α = .82). Data analysis involved Independent-samples t-test for the practical test administered at the end, MANOVAs with repeated measures to compare the pre- and post-tests results of the two groups for all the matched data (pre-post).

Qualitative data analysis was used to examine students’ perceptions of the simulated learning environment, elicited in an open-ended question format at the end of the clinical skills practical test. The analysis was also used to examine peer and SPs perceptions on the students’ performance during the practical skills test. The free text responses were manually coded, and thematic analysis of the data was undertaken to identify patterns and dominant themes [31].

## Results

### Comparing the effect of a simulation-based learning with a peer-based learning

The means and standard deviations for all measures used in this study are displayed in Table 4, and statistical tests were carried out to test the four hypotheses.

**Table 4:** **Pre- and post-means and standard deviations for all measures (Practical exam, clinical skills self-confidence, engagement in lifelong learning, and clinical knowledge test)**

**Assessment instrument and group**	**N**	**Time**	**Mean**	**Std. Dev.**	***p***
Practical Exam	43	2	11.97	4.62	
Peer-patient	22	2	10.18	3.78	
Standardised patient	21	2	13.47	4.91	.018*
Pre-Clinical skills self-confidence	43	1	2.71	1.06	
Peer-patient	22	1	2.56	.963	
Standardised patient	21	1	3.60	.694	
Post-Clinical skills self-confidence	43	2	3.97	.897	.000*
Peer-patient	22	2	2.87	1.15	
Standardised patient	21	2	4.40	.897	
Pre-Engagement in lifelong learning	43	1	3.11	1.08	
Peer-patient	22	1	2.77	.956	
Standardised patient	21	1	3.44	1.13	
Post-Engagement in lifelong learning	43	2	3.53	1.09	.003*
Peer-patient	22	2	3.12	.963	
Standardised patient	21	2	3.93	1.09	
Pre-Clinical knowledge test	43	1	4.88	2.77	
Peer-patient	22	1	5.04	2.73	
Standardised patient	21	1	4.71	2.88	
Post-Clinical knowledge test	43	2	11.88	2.70	.000*
Peer-patient	22	2	11.50	3.05	
Standardised patient	21	2	12.28	2.28	

*significant at *p* < 0.05.

Hypothesis 1 related to clinical skills was supported. An independent-samples t-test revealed a significant difference between the group of students who worked with simulated patients (M = 13.47, SD = 4.91) compared to those who worked with peer patients (M = 10.18, SD = 3.77); t(41) = -2.47, p =0.018. These results reveal that students who worked with a standardised patient displayed higher standards of clinical skills than those who worked with peer patients.

The null hypothesis (Hypothesis 2) related to the development of clinical knowledge was also supported. A MANOVA for group by time for the clinical knowledge test revealed no interaction effect. Both groups improved their clinical knowledge over time (F = 189.7 (1, 31), p = 000).

Hypothesis 3 related to the development of self-confidence in clinical skills was not supported. A MANOVA for group by time for the self-confidence test revealed no interaction effect. Both groups displayed significant overall improvement in self-confidence overtime (F = 56.91 (1, 40), p = 000). Table 4 displays the means and standard deviation for these measures.

Finally, Hypothesis 4 related to motivation (engagement) for lifelong professional development in the area of musculoskeletal rehabilitation was not supported. A MANOVA for group by time for the motivation scale revealed no interaction effect. Overall both groups displayed greater motivation for lifelong professional development overtime (F = 10.05 (1, 40), p = .003).

### Exploring students’ perceptions of the simulated learning environment

All 21 students who were assigned to the SPs condition responded to the open-ended question eliciting their perceptions and experience of this learning environment. Five main themes emerged from the qualitative data analysis of their accounts. There were three positive themes, namely, preparation for professional practice, exposure to different body types and positive challenge and two negative themes, titled, lack of feedback, and stressful situation. Table 5 displays these themes.

**Table 5:** **Students’ perceptions of standardised patients (SPs)**

**Themes**	**Examples from the data**
***Positive***	
*Preparation for professional practice*	*Provided a sense of being in a real clinical setting*
*Exposure to different body types*	*She (patient) had an injury*
*Positive challenge*	*Think on our feet*
	*Put in a situation to test our knowledge*
***Negative***	
*Lack of feedback*	*They didn’t know what I was testing so they couldn’t pre-empt or hint at*
*Stressful situation*	*It made me take it* (lab) *more seriously which was quite nerve racking*

More than half of the group (17/21) reported positive aspects and of these, 13 conveyed both positive and negative attributes. On the positive side, the prevailing theme centred on providing them with a real-life scenario which can carry over into the professional setting. More specifically, students mentioned preparation for professional practice, exposure to various physical characteristics, and the challenging aspect of working with a standardised patient, which participants felt provided them with a better simulation of professional practice. For example,

#### Preparation for professional practice

A majority of participants (13) expressed the SPs experience as having a positive impact for their future as a professional, reporting that they felt it better prepared them for practice:

*“I liked the feel of assessing someone I didn’t know creating a real-life patient/provider scenario (which) will help in building confidence and experience which will carry on in further careers”* (student 13)

*“….It gave me a sense at being in a real clinical setting”* (student 40)

*“I liked that it [standardised patient] forced me to concentrate more and act more professionally”* (student 17)

#### Exposure to different body types

A few participants (5/21) noted the valuable experience of working with an unknown population, that is, people who were of different age, physical health and body morphology.

*“Got to work on different body types”* (student 3)

*“I liked that she* (SP) *had an injury that I could use to test on”* (student 25)

*“Liked different body which allowed me to look objectively at a stranger”* (student 28)

#### Positive challenge

Six participants felt that the experience was a challenge but this was viewed with positive responses.

*“It was a lot harder and scarier doing it on someone I didn’t know which was very good”* (student 19)

*“Allowed us to think on our feet*” (student 28)

*“…Liked the fact I was put in an uncomfortable situation to test our knowledge”* (student 5)

Negative experiences or disliked aspects varied, with a minority of students (4/21) expressing exclusively a negative experience or a dislike of some specific aspect. Their comments were similar to those students who reported both positive and negative experiences. The two negative themes are presented below, with direct quotes to illustrate students’ accounts of their experience.

#### Lack of feedback

The most common negative theme was lack of patient feedback, which was reported by a third of the group (7/21). These students thought that due to the SPs being “trained to perform”, they had limited or no knowledge about the condition and evaluation procedures. Consequently, learning was not as effective as working with a peer, with whom ideas could be freely discussed.

*“There was no feedback and communication so it was hard to know if I was doing it* (skill) *correctly”* (student 16)

*“…you couldn’t bounce ideas off them* (peer) *or ask them questions”* (student 3)

*“They didn’t know what I was testing so they couldn’t pre-empt or hint at what we should do”* (student 28)

#### Stressful situation

Almost half of the group (9/21) mentioned the increased level of stress raised by working with SPs. Students expressed concerns about making mistakes and the professional atmosphere of the lab. As all these participants were familiar with the use of peer-patients through previous, unrelated units, the use of SPs exposed them to an environment, which was perceived as less comfortable.

*“It made me take it* (lab) *more seriously which was quite nerve racking”* (student 9)

*“I disliked having a standardised patient which seemed to put considerable more stress and pressure on me”* (student 29)

*“I found it added to my nerves because I know that they were older and this made me nervous. I feel more comfortable with a peer; I am less inclined to be scared if I’m making a mistake with a peer”* (student 36)

### Exploring standardised and peer patients’ perceptions of students’ performance

All students’ clinical skills were assessed with a practical test, performed with either SPs or PPs, depending on the group they had been assigned to. After each student had been assessed the SPs and PPs were invited to respond to an open-ended question on their perceptions of the students’ professional and communication skills performance during the clinical skills assessment (this feedback was not provided to the student at the time of the test). Specifically, they were asked to describe how the student appeared professionally and while communicating, while assessing them. All PPs and SPs reported on their respective student’s performance. Table 6 displays these themes.

**Table 6:** **Standardised and peer patients’ perceptions of students**

**Themes from standardised patients (SPs)**	**Examples from the data**
***Positive***	
*Confident*	*Seemed quite confident*
*Comfortable*	*The student appeared relaxed and comfortable*
***Negative***	
*Uncomfortable*	*Had some nervous moments where he lost his train of thought and professionalism*
**Themes from Peer Patients (PPs)**	**Examples from the data**
***Positive***	
*Relaxed*	*Student looked relaxed, and did not appear too nervous*
***Negative***	
*Uncomfortable*	*Student was very nervous. He let that get the better of him*
	*Not very comfortable and was fairly nervous and unsure*

From the SPs perspective, two positive themes emerged, namely, being confident and comfortable, and one negative theme, namely, feeling uncomfortable.

Out of the 21 students in the SP condition, 14 were only perceived positively and one only negatively. The other six were perceived in both positive and negative terms. For example,

*“Student was confident and relaxed but nervous. Acted professional”* (student 8)

*“Seemed quite confident, however had some nervous moments where he lost his train of thought and professionalism”* (student 13)

*“The student appeared relaxed and comfortable with me and did not appear too nervous on the finding of pain or positive tests”* (student 22)

Within the PPs condition, one single positive theme emerged, namely relaxed, with one negative theme, uncomfortable being the overriding factor. Out of the 22 students in this condition, none were perceived only positively, nine were perceived only negatively while the remaining (13) were perceived in both negative and positive terms. For example,

*“A bit stressed at first but then seemed to relax towards the end”* (student 4)

*“Not very comfortable and was fairly nervous and unsure”* (student 11)

*“Student was very nervous. He let that get the better of him. Because he was nervous it affected his performance, e.g., remembering what tests to do. Could tell he was uneasy”* (student 31)

*“Student looked relaxed, and did not appear too nervous, but did go through the exam a bit quick though”* (student 33)

The dominantly positive perceptions provided by the SP (20/21, 14 of them exclusively positive) in comparison to the PP (13/22, none of them exclusively positive) suggests that although both groups did appear to be nervous and stressed, more than likely due to this being an assessment which formed their final mark, those students in the SPs condition may have developed a greater sense of professionalism which was reflected in their performance. This building sense of professionalism, which may have developed over the two-week lab sessions, may have a direct effect on how students perceive the importance of practice laboratory sessions. This, in turn, may have resulted in them practising their skills more intently.

Overall, these results demonstrated that the use of SPs is beneficial for the development of clinical skills. However, some aspects of the simulated learning environment, especially working with unknown people, can place some students in a position of discomfort and hinder their development of confidence and in turn their desire to engage further in the area being taught. Students and patient actors’ (PPs and SPs) comments also highlight the importance of comprehensive preparation of the SPs; especially sufficient understanding of the procedures students had to practise.

## Discussion

The use of SPs in musculoskeletal curricula was explored in this study. As with health professions programs such as medicine and exercise physiology, educators are challenged to prepare students entering the profession to be skilled in evaluation, critical thinking, self-analysis, and decision making in musculoskeletal assessment and rehabilitation. The results from this study provided support for the learning value of implementing a simulated learning environment, more specifically the use of SPs in a musculoskeletal rehabilitation course, to increase students’ development of clinical skills.

This form of instruction may contribute to bring students’ knowledge and skills closer to other health professional education programs, which have scope within their curricula to incorporate multiple units in this specialised area. For example, approximately one third of physical therapist education programs in the United States utilise SPs [32] and have multiple units in musculoskeletal evaluation and rehabilitation. While this is a vital component of physical therapy (physiotherapy) there are similarities in the practice of musculoskeletal rehabilitation in exercise physiology. We would argue that exercise physiology, like medical education programs more generally, need to include pedagogical methods that adequately prepare students for this professional challenge. Yet, if programs are already crowded, multiple class/unit/module in musculoskeletal evaluation and rehabilitation cannot be offered, thus alternative, time-effective approaches need to be considered.

Whilst a few previous studies had reported positive results with the use of SPs [33-38], the outcome measures were mostly isolated to one skill, or elicited subjective feelings such as self-confidence or anxiety of the students. In contrast, the present study measured the actual outcome of an evaluation to an area of the body, which required knowledge and the ability to perform a variety of shoulder physical evaluation skills.

In this study, the lack of impact of working with a standardised patient compared to working with a peer as patient, on students’ clinical knowledge (written test), self-confidence in clinical skills and enhanced motivation for lifelong learning can be interpreted in terms of the one-off intervention with very limited exposure to SPs. In light of previous research support for the use of SPs in enhancing areas such as communication skills, and self-confidence [21-23], future research should consider longer timeframe (e.g. a full semester) and several exposures to SPs. It should also compare musculoskeletal knowledge and skill to health professional education programs that have several classes/units/modules in this area in order to determine if it is can be an alternate method of instruction.

In regard to the lack of increase in motivation (engagement) for lifelong professional development, and self-confidence in clinical skills, it is possible that students who worked with SPs, who were people they did not know and who were trained to respond to pain during treatment, may have had their self-confidence and motivation challenged more so than those who worked with supportive, familiar peers. This was captured in the feedback from those students who were exposed to SPs. For example, a few reported the experience being *“very nerve racking”* and “…*more stress* [ful]*”*, whilst others reported a lack of feedback such as “*I couldn’t discuss things I was confused about with my peers”* as potential challenges to their confidence. However, it must be noted that the SPs were instructed to provide constructive feedback to students about their professional and communication skills and not how well they performed the skill (they are after all, acting as patients with limited knowledge of the skills being applied). This was clearly the case as students wanted the SPs to provide feedback on the skills as was noted by this student, “*They didn’t know what I was testing so they couldn’t pre-empt or hint at what we should do”.* Even though students perceived their self-confidence as being challenged it may not have shown outwardly in appearance. This was reported in the feedback from the SPs. For example, “*Seemed quite confident however had some nervous moments where he lost his train of thought…”* This is in comparison to the PP group who received more negative comments categorised as them being uncomfortable in appearance. The contrasting outwardly appearance of high confidence with a reduced level of self-perceived confidence is important as increasingly more research is being paid to patients’ views about their doctors (and their appearance of confidence) and the relationship between health outcomes [39] such as reducing anxiety levels and preventing possible psychological complications [40].

Whilst possessing optimal levels of self-confidence in clinical skills may be ideal [41,42], the extent to which self-confidence reflects actual competence in clinical skills is contentious. Some research has revealed a lack of direct relationship between self-confidence and competence [43,44], leading some to conclude that self-confidence may not be a reliable indicator of actual competence [45,46]. However, possessing optimal levels of self-confidence in skills is important because it is a self-evaluation of competence and capability to effectively manage various situations. As such, this provides motivation, which is a key determinant of persistence in difficult learning activities [47]. In part, this link between optimal levels of self-confidence and increased motivation to practice and apply learnt skills [48], has contributed to the view that self-confidence is a central component in effective clinical performance [49] and therefore may impact motivation for lifelong professional learning.

Important to educators are those labs which use PPs scenarios which may lack the challenges of practicum and eventual practice as expressed by one student, “*I feel more comfortable with a peer; I am less inclined to be scared if I’m making a mistake with a peer”.* Therefore it may be important, whilst designing labs that utilise PPs, to incorporate components such as limited feedback, and less compliance with the peer-patient.

The importance of feedback cannot be overlooked, as the information provided by an agent (e.g., teacher, peer, and patient), is vital for improving one’s performance and understanding. It can increase effort, motivation, or engagement and/or it can increase cue searching and task processes, which in turn lead to understanding (motivation to engage in further learning). Feedback is well established as having among the most critical influences on student learning [50]. Kluger et al. [51] concluded that feedback is effective to the degree to which it directs information to enhanced self-efficacy (self-confidence) and to more effective self-regulation, such that attention is directed back to the task and causes students to invest more effort or commitment to the task. They claim that such feedback is likely to yield impressive gains in performance. This needs to be highlighted when programs use SPs as there needs to be a feedback mechanism in which the student can adequately learn and seek further help.

Age may play a role in how well SPs are able to contribute to effective education. This was brought up with the SPs age difference which spanned from 22 to 40 with a majority in their mid to late 20s. This was in contrast to the PP group, which had 34 students in the 18-22 age range, 7 in the 23-28, and 2 over the age of 28. This was reflected in a student’s comment, *“I found it added to my nerves because I know that they were older and this made me nervous”.* This was an interesting aspect which emerged and is not documented in the literature.

From an educator’s perspective, both peer role-play and SPs represent valuable tools for clinical knowledge, self-confidence and enhanced motivation for lifelong learning. The positive effect of both methods is noteworthy as it suggests that students can benefit from both learning opportunities but in different ways. Peer role-play, whilst perhaps less sophisticated than the application of SPs, nevertheless offers highly valuable training scenarios with the opportunity for peer feedback. Research has also explored the association between various standardised patient types and students’ perceived learning experiences. Mavis et al. [52] specifically looked at the use of actors, peer students, and instructors as standardised patients in performance-based assessments. They reported that students were feeling most intimidated by instructors and least nervous with peers. Instructors gave the best feedback, whilst actors were found to be most believable as a patient. Despite these differences, Mavis et al. [52] found that their students were generally confident in their simulated diagnosis, did not feel their performance was inhibited, and had a positive learning experience with each type of standardised patient utilised. This finding is also consistent with our students’ perceptions of their confidence in skill development with both patient types (peers and actors).

The use of SPs in examination skills training, however, remains a more powerful tool than peer role-play and therefore both warrant inclusion in health professional education curricula. Peer role-play constitutes a valuable tool for undergraduate clinical skills training, since it requires few resources and most importantly because it allows students to personally experience what a patient encounters during a physical assessment procedure. Conversely SPs may have a greater impact on the development of clinical skills but given the expense, time and resources, they may not be used on a regular basis, peer role-play being a more affordable and highly suitable alternative [53]. Future research may also explore the benefits of a staged approach, carried over an extended period of time, starting with peers and following up with semi-formal SP situations. This would allow early exposure with peers so they get comfortable with the process and obtain informal feedback, share ideas and information, and then later with SPs.

While the use of SPs can be an added expense, the cost may be reduced if other approaches are considered. One possibility may be to seek volunteer participants who live in retirement villages. This option may have mutual benefits, since it would offer a way to engage a community of retirees in students’ learning process while at the same time exposing students to a population that they will encounter in great numbers whilst in professional practice. The use of research participants in other studies could be another way to attain standardised patients. This would be accomplished by implementing a learning component in a research project, for example, by asking participants being assessed for cardiovascular, strength, balance and fitness levels in other research to contribute as a standardised patient in a related course (i.e., cardiovascular rehabilitation, fitness assessment, etc.).

Limitations to this study include the small number of participants and relatively short lecture and lab time frame. A longer intervention may have yielded different results. Also, the basis for assigning students to groups did not include level of clinical practical skills, which were assessed only after the lecture and lab sessions. It was decided that this would be difficult as the students did not have any prior practical skills experience with this material and therefore may not have accurately self-assessed their proficiency. However, all students did have prior theoretical knowledge, which we felt would be a better way to assign them to groups. Students’ reports of their clinical self-confidence and their engagement in lifelong learning were two other elements not used to assign students to a particular condition. Taking into account these two aspects may have generated groups with different profiles and in turn different findings. This should be explored in future research.

## Conclusion

The present study examined the benefits of training practice in musculoskeletal assessment and rehabilitation using SPs in a short time frame. Despite limitations such as small sample size, a two-week lecture and lab time frame, limited exposure to SPs and lack of qualitative data from students who practised with a peer, the findings provide support for the value of using SPs to enhance students’ clinical assessment skills in musculoskeletal rehabilitation, and more generally to prepare health professional students to meet the challenge of patient needs. Specifically, the findings suggest that the use of SPs could play a vital role in achieving optimal levels in knowledge and skill in health professional programs that can only incorporate a few modules in the area of musculoskeletal assessment and rehabilitation. Overall, the main barrier related to the use of SPs remains the financial aspect but once a pool of SPs has been constituted, the overall costs to a study program would decrease. Considering other novel approaches to attain participants willing to act as patients may represent an alternate, yet effective, approach in attaining a pool of volunteers on a continual basis.
